# Potential Valorization of Hazelnut Shells through Extraction, Purification and Structural Characterization of Prebiotic Compounds: A Critical Review

**DOI:** 10.3390/foods10061197

**Published:** 2021-05-26

**Authors:** Andrea Fuso, Davide Risso, Ginevra Rosso, Franco Rosso, Federica Manini, Ileana Manera, Augusta Caligiani

**Affiliations:** 1 Food and Drug Department, University of Parma, Via Parco Area delle Scienze 17/A, 43124 Parma, Italy; augusta.caligiani@unipr.it; 2 Soremartec Italia Srl, Ferrero Group, 12051 Alba, Italy; davide.risso@ferrero.com (D.R.); Ginevra.ROSSO@ferrero.com (G.R.); franco.ROSSO@ferrero.com (F.R.); federica.MANINI@ferrero.com (F.M.); ileana.manera@ferrero.com (I.M.)

**Keywords:** hazelnut shells, xylooligosaccharides, arabino-xylooligosaccharides, XOS, AXOS, circular economy, prebiotic, functional foods

## Abstract

Hazelnuts are one of the most widely consumed nuts, but their production creates large quantities of by-products, especially shells, that could be upcycled into much more valuable products. Recent studies have shown that hazelnut shell hemicellulose is particularly rich in compounds that are potential precursors of xylooligosaccharides and arabino-xylooligosaccharides ((A)XOS), previously defined as emerging prebiotics very beneficial for human health. The production of these compounds on an industrial scale-up could have big consequences on the functional foods market. However, to produce (A)XOS from a lignocellulosic biomass, such as hazelnut shell, is not easy. Many methods for the extraction and the purification of these prebiotics have been developed, but they all have different efficiencies and consequences, including on the chemical structure of the obtained (A)XOS. The latter, in turn, is strongly correlated to the nutritional effects they have on health, which is why the optimization of the structural characterization process is also necessary. Therefore, this review aims to summarize the progress made by research in this field, so as to contribute to the exploitation of hazelnut waste streams through a circular economy approach, increasing the value of this biomass through the production of new functional ingredients.

## 1. Hazelnut and Circular Economy

*Corylus avellana* L., the European hazelnut, whose production ranges from North Africa and Europe to Asia Minor and the Caucasus region, is the second most popular nut worldwide just after almonds. Its crop added up to over 528,000 metric tons (kernel basis) in 2019/20. Turkey is the main producer and exporter, with 63% and 66% of the global production and exports, respectively, followed by Italy, accounting for 13% and 11% of the share. Azerbaijan, Georgia, and the USA are the other shelled hazelnut producers [1]. Among the producing countries, hazelnut consumption per person and per year is highest in Italy (0.520 kg kernel/person) followed by Greece (0.369 kg kernel/person) and Turkey (0.250 kg kernel/person). Worldwide, the highest hazelnut consumption per person and year is reached in Switzerland (2.096 kg kernel/person). These nuts are typically consumed whole (raw or roasted) or used as an ingredient in a variety of foods, especially in bakery and confectionery products. Moreover, hazelnut oil is also used as a cooking oil. They are highly appreciated for their organoleptic properties and are also very nutritious and healthy due to their favourable nutritional composition. A study carried out on 17 different varieties of hazelnut cultivated in Turkey, in fact, reported a moisture content of 2–5%, a lipid content of 56–68% (mainly mono- and polyunsaturated), protein content of 12–21%, and 2–3% of ash [2]. In addition, dietary fibre plays a fundamental role in the nutritional profile of hazelnut, with a reported content of 10–13% on dry matter, the majority of which is water-insoluble [3,4], even if other authors have attributed to it lower contents, between 7% and 8% [5,6], or higher ones, up to 18% [7]. Such broad variation may be explained, at least in part, by the studied cultivar and by the level and type of processing the hazelnuts are subjected to (e.g., natural, roasted, etc.). Hazelnuts are also good sources of micronutrients and bioactive compounds, in particular of natural antioxidants. While they represent one of the best sources of vitamin E among tree nuts, they are also good sources of vitamin K, phosphorus, calcium, and magnesium, among others [4,8]. In addition, hazelnut skin has been shown to be one of the highest dietary sources of polyphenols, in particular of flavan-3-ols in both their monomeric and polymeric forms [9]. One of the main drawbacks associated with hazelnut production is the large amount of by-products. In fact, hazelnuts are collected in the form of dried in-shell nuts that are further processed to be introduced into the industrial food chain. The residual biomass resulting from the cracking process, the hazelnut shell (HS), represents approximately 50–55% of the weight of the in-shell product and today is mainly used as a boiler fuel.

In recent years, the world has had to face the risk of destroying global ecosystems, and consequently there has been a marked increased interest in issues such as environmental sustainability, waste limitations, and application of circular economy principles. To achieve these goals, it is necessary to decouple economic growth from resource consumption. The old “take, make, dispose” linear production approach focused on exploiting resources is no longer viable and has therefore been replaced by the concept of circular economy, which while not entirely new, has recently gained importance both in policymakers’ agendas and in the activities of non-governmental organizations (NGOs) and private companies. However, the circular economy is something more than recycling waste, it means transforming what was considered waste into high-value resources. In March 2015, the EEA published “The European environment—State and outlook 2015 (SOER 2015)” [10], which provides a comprehensive assessment of the European environment and sets it in a global context. It informs European environmental policy implementation and analyses the opportunities to achieve the EU’s 2050 vision of “living well within the limits of the planet”.

In this context, many researchers focused their work on the valorisation of the agri-food leftovers as functional ingredients for novel food production, and the possibility of using hazelnut by-products as an alternative source for bio-active ingredients/additives has emerged. In fact, the HS are potentially very precious, abundant, and cheap by-products from which high-added value ingredients can be obtained [11]. Their main constituent is lignin, with quantities found around 40–51%, followed by hemicellulose with 13–32%, and cellulose (17–27%) [12,13,14,15,16,17]. Like all lignocellulosic biomasses, HS have such a complexity, due to the polymeric nature of their components, that their fractionation is almost inevitable. In this sense, this by-product fits perfectly in the world of biorefinery, which is based on the selective separation of the main components of raw materials, converting them into new materials, chemicals, and energy [18]. The production of chemicals is expected to be derived increasingly from plant biomass [19], and lignocellulosic biorefineries will be a key component of the industrial sector of the near future. They will ensure sustainability through the conversion and reuse of raw materials deriving from agriculture and forestry [20]. In recent years, scientific research has focused heavily on studying different ways to exploit lignocellulosic biomass, including HS. In fact, today, the latter are used almost exclusively as boiler fuel for heat recovery [11], but the effective valorisation of a by-product depends on the capacity to guess its maximum potential. In this sense, several researchers have proposed using HS by exploiting its high lignin content, transforming it into activated carbon which could then be used for the adsorption of heavy metals from the environment, such as lead [21,22], chromium, cadmium, zinc [23,24], nickel [25], copper [26], arsenic [27], but also dyes [28] and CO_2_ [29]. Other studies have investigated the possible use of HS to produce hydrogen [30,31], ethanol [32], renewable fuels and chemical feedstocks [33], or fibreboards [34], but also to exploit its content in phenolic compounds [35,36,37,38]. However, in recent years many studies have focused their attention on the hemicellulosic fraction extracted from HS, pointing out the presence of very interesting compounds that could reveal new possible uses of this by-product. In fact, the hemicellulose in HS is largely composed of xylans (often substituted with uronic acids and acetyl groups), as well as smaller quantities of arabinans and galactans [14,15,17,39]. Xylans have potential as a feedstock for the production of xylooligosaccharides (XOS) from autohydrolysis [14]. In addition, another type of oligosaccharides (OS), namely the arabino-xylooligosaccharides (AXOS), have been identified in HS after the extraction of hemicellulosic fraction [40], although it is not well known what is the amount of XOS and AXOS, respectively.

## 2. Chemical Structure of (A)XOS

**Figure 1:**
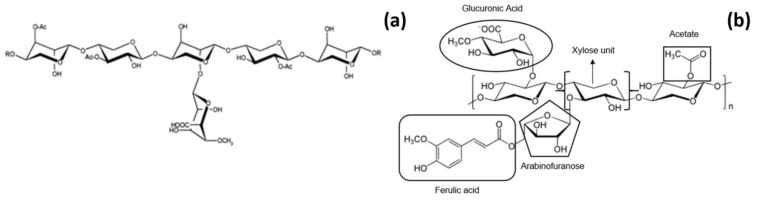
(**a**) Xylooligosaccharide extracted from hardwood. Source: Vázquez et al., 2000 [45]. Reproduced with permission from Vàzquez et al., Trends in Food Science & Technology; published by Elsevier, 2000. (**b**) Arabino-xylooligosaccharide showing different bonds and substitutions. Source: De Freitas et al., 2019 [46]. Reproduced with permission from De Freitas et al., Bioactive Carbohydrates and Dietary Fibre; published by Elsevier, 2019.

(A)XOS are a mix of OS constituted by a linear β-(1→4)-D-xylopyranan backbone. In general, the oligomers that contain from 2 to 10 molecules of xylose are considered XOS (or AXOS, depending on whether D-arabinose is present as substituent or it is not), even if some authors include in this category also molecules with degree of polymerization (DP) ≤ 20 [41]. (A)XOS can be obtained from main-chain-xylan compounds and their precise chemical structure (Figure 1) varies according to the extraction process and to the source they are obtained from. (A)XOS may contain different substituents bonded to the xylose-based backbone, such as acetyl groups, phenylpropenoic acids (like hydroxycinnamic acids, mainly ferulic acid, and to a lesser extent dehydrodiferulic acid, p-coumaric acid, and sinapic acid), uronic acids (like α-D-glucopyranosyl uronic acid or its 4-O-methyl derivative), other xylopyranose units and so on, and this makes their structure branched [41,42,43]. When α-L-arabinofuranosyl is one of the substituents, we are talking about AXOS: this monosaccharide is linked to the backbone with α-1,2 and α-1,3 glycosidic bonds [44]. The degree of substitution between arabinose and xylose is known as A/X ratio. Therefore, xylose molecules can be unsubstituted (XOS), monosubstituted with a single α-L-arabinofuranoside at either C2 or C3, or disubstituted with single α-L-arabinofuranoside units at C2 and C3 [44].

## 3. A(XOS): Emerging Prebiotics

**Figure 2:**
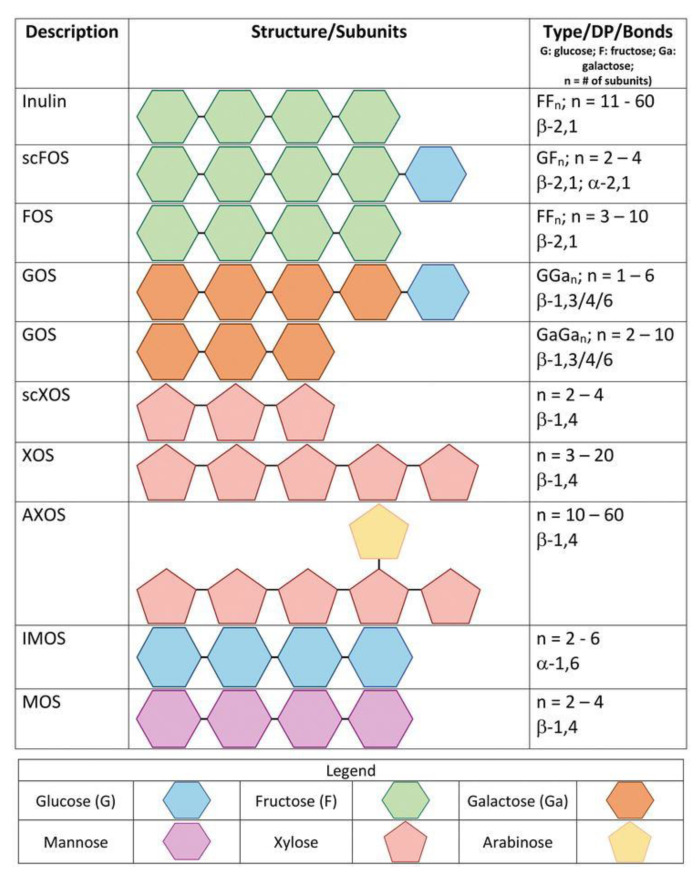
Different chemical structures of prebiotics available in the market. Source: Saville and Saville, 2019 [59]. Reproduced with permission from Saville and Saville, Prebiotics and Probiotics—Potential Benefits in Nutrition and Health; published by IntechOpen, 2019.

Consumers are increasingly aware of the relationship between diet and health and for this reason trends in food choices are changing [47]. Indeed, with onsets of chronic obesity, gastrointestinal diseases, diabetes, coronary diseases, cancers, and degenerative diseases on the rise, the trend in consumer choices is increasingly moving towards prebiotics, probiotics, and functional foods more in general, with the market for these products constantly expanding [48]. In fact, the global prebiotic ingredients market was estimated at $4.07 billion in 2017, and the forecast is that it will reach $7.37 billion by 2023, registering a compound annual growth rate (CAGR) of 10.4% [49]. For these reasons, the optimization of the extraction and characterization techniques of compounds such (A)XOS would be advantageous. Prebiotics have been defined as “nonviable food components that confer a health benefit on the host associated with modulation of the microbiota” [50]. Such a prebiotic effect is associated with an increase in selective bacteria groups that exert beneficial effects on the gastro-intestinal health of the host, such as bifidobacteria, lactobacilli, and eubacteria, with a consequent inhibition of potentially harmful bacteria, such as enterobacteria and clostridia [51]. Prebiotics have a therapeutic potential still to be discovered; however, it is very promising for the inflammatory bowel disease (IBD) [52] and colon cancer treatment [53], for the protein fermentation in the colon [54], for lipid, energy, and glucose metabolisms, mineral absorption and stool transit [44]. Furthermore, gut microbiota are strictly associated with cardiovascular diseases [55], obesity [56], and type II diabetes [57], therefore their modification through an adequate diet could have dramatic positive effects on all these conditions [44]. The prebiotics more commonly found in the market are fructans such inulin and fructo-oligosaccharides (FOS), although galacto-oligosaccharides (GOS), resistant dextrins and starches (RS), mannooligosaccharides (MOS), and isomaltooligosaccharides (IMOS), among others, are also available commercially: their different structures are represented in Figure 2. AXOS and XOS are considered as promising emerging prebiotics, even though the latter have been available in Japan since the 1980s [42,44,58,59].

To be included as prebiotic, a substance needs to meet some requirements, scientifically demonstrated: it must be resistant to gastric acidity, to digestive enzymes, and to gastrointestinal tract absorption, it must be fermented in the colon and be able to stimulate the selective growth of microbial populations that beneficially affect the host health [60]. The congruence between (A)XOS and these requirements has been discussed in detail in the review of Broekaert and colleagues [44]. First, it has been shown that AXOS have greater acidity resistance than FOS and no mammalian enzyme capable of hydrolysing (A)XOS has been found [44,61]. As regards fermentability in the colon of (A)XOS, this has been confirmed by several in vitro [62,63] and in vivo studies [64,65]. Finally, Vardakou et al. found that wheat-derived AXOS allowed the growth of positive microbial populations, and in particular *Bifidobacterium* spp levels [66]. The bifidogenic effect of AXOS intake, together with an increase of butyrate producers in the gut microbiota, has also been reported by a recent randomized cross-over trial [67]. In addition, it was observed that XOS allowed the growth of a larger quantity of *Bifidobacterium* strains compared to AXOS [44]. Several studies have also highlighted the prebiotic effects of XOS with higher selectivity for bifidobacteria, in particular, pointing to their potential role as functional ingredients in a variety of food products [42,68,69].

### 3.1. Potential Health-Related Effects of (A)XOS

Many clinical studies have focused their attention on the prebiotic effectiveness of (A)XOS, although only recently and in short-term trials. For this reason, it is still necessary to confirm the replicability of such results and further verify the many beneficial effects that have been attributed to (A)XOS consumption. Many clinical studies have focused their attention on the prebiotic effectiveness of (A)XOS, although only recently and in short-term trials. For this reason, it is still necessary to confirm the replicability of such results and further verify the many beneficial effects that have been attributed to (A)XOS consumption. For instance, different studies about XOS have been conducted on rats, showing their potential in diabetes prevention, through the improvement of metabolic parameters [70]. Other studies laid the foundations to establish a possible beneficial effect of XOS on oxidative stress [71] and colon inflammation [69]. Lipid metabolism also seems to be affected by a XOS-rich diet, but conflicting results have been obtained. In fact, some studies reported a decrease in blood cholesterol and triglycerides as well as an increase in faecal triglycerides and cholesterol, both in rats [72] and in humans [73], but this did not occur in the study conducted by Chung et al. with a comparable dose of XOS [74]. As regards other trials on humans, in a Japanese study conducted on adult women, stool frequency and abdominal conditions were significantly improved by XOS treatment [75]. Chung et al. performed a study with people over 65; their results highlighted a change in stool characteristics, with a higher moisture content and a lower pH, after a period of three weeks consuming XOS. Mineral absorption and blood parameters did not show significant variations compared to the control [74]. Moreover, also the property of preventing diabetes, through the modulation of gut microbiota, has been attributed to XOS [76]. Another advantage of XOS is their non-cariogenicity since they are not used by oral microbiota [45]. Finally, it seems that they also have antiallergic activities and beneficial effects on the skin [77].

As for AXOS, so far mainly extracted from cereals, many and similar prebiotic properties have been found recently. First of all, they as well as XOS promote the growth of bacteria belonging to *Bifidobacterium* genus and induce an increase in the short chain fatty acids (SCFA) production [78,79,80]. As regards lipid metabolism, some studies agree pointing out that no significant modifications exist concurrently with an AXOS-based diet [67,78,81]. These compounds are also associated with attenuations in type II diabetes [82], as well as improvements in immunomodulatory activity [83]. Then, AXOS consumption seems to promote a greater insulin sensitivity, going to improve glucose metabolism [80,84].

### 3.2. Recommended Dose and Exposure Assessment for (A)XOS

(A)XOS are naturally present in a variety of foods, such as cereals, honey, bamboo shoots, fruit, and vegetables, but their concentration is often too low to allow the beneficial effects onset [42,78]. All the in vivo studies that achieved positive results in terms of XOS prebiotic efficiency provided absolute quantities varying from 1.3 to 3.9 g/day [44]. This threshold value is a far lower compared to that of other prebiotics available today and this makes XOS really competitive on the market [49]. Some considerations have been made also in terms of possible undesired effects associated with excessive quantities. This topic has been discussed in depth by EFSA’s scientific experts following an initial assessment made by the competent authority of Hungary, after receiving a request to place a xylo-oligosaccharides (XOS) mixture obtained from corncobs by enzymatic hydrolysis on the market as a novel food (NF) in 2018 [85]. In this scientific opinion, EFSA reported that acute toxicity studies have been performed in mice and dogs at high XOS doses. Soft stools, vomiting, and diarrhoea were the adverse effects observed at doses of 6–14 g/kg body weight in dogs and 5–10 g/kg body weight in rats, but in both cases the animals recovered after one or two days [85]. No mortalities occurred in rats even when XOS dose was increased to 32 g/kg body weight [86]. Regarding clinical trials on humans, Xiao et al. provided volunteers 10–12 g of XOS per day, observing a diarrhoea onset in the first day of consumption among 18% of the subjects, compared to 6% of the control. However, after one week test no adverse effects occurred anymore [87]. The same observed phenomenon happened in another study, at the dose of 10 g XOS per day [88]. In the scientific report issued by EFSA in 2018, it has been stressed that the aforementioned adverse effects were also associated with the consumption of other non-digestible carbohydrates. For all these reasons, the European Commission finally agreed to add XOS deriving from corncobs and treated with xylanases to the novel foods list. The maximum use level varies, depending on the product, between 0.35 and 3%. More specifically, in milk, milk product imitations, and fermented milk products, the maximum use level equals 0.35%; in bread, rolls, and fine bakery wares 1.4%; in jams, marmalades, fruit spreads and chocolate 3%. Based on these premises, the EFSA Panel concluded that such NF, a mixture of XOS, is safe under the proposed uses and use levels for the general population [85]. This led the European Commission to authorise the placing on the market of xylo-oligosaccharides as a novel food [89]. Subsequently, in July 2020, such XOS mixtures have also been authorized for use in dietary supplements at a maximum dose of 2 g/day [90].

As regards AXOS, their prebiotic efficiency has been demonstrated for similar quantities to those of XOS. Cloetens et al., for example, showed positive effects onset after AXOS consumption at a dose of 2.2 g/day [91]. Moreover, to date, in no clinical study has the use of AXOS caused adverse health effects, except for mild flatulence when doses reached 10–15 g/day [45]. For this reason, AXOS were added in 2017 by the European Commission to the novel foods list when they are obtained from wheat bran extract through enzymatic extraction. The addition of these compounds is regulated in maximum doses ranging between 0.4 and 9 g/100g product and this functional ingredient must be at least 70% (expressed in dry weight) AXOS [92].

## 4. Extraction 

The first step for prebiotic functional foods creation starting from HS is obviously the extraction of (A)XOS. Recently, several studies have focused their work on the development of a method to optimize the extraction yield, starting from different lignocellulosic/lignohemicellulosic materials and not only (Table 1). Different chemical structures of raw materials and different process conditions may however affect the composition and structure of obtained OS, also affecting their functional properties [14]. Moreover, the sample pre-treatment is a very important operation. First of all, physical pre-treatments, such as grinding, are useful for reducing the particle size and increasing the contact surface area of lignocellulosic material [93]. 

### 4.1. Autohydrolysis Treatments

After the physical one, other kinds of pre-treatments are essential for degrading lignin structure, thus allowing hemicellulose exposure to hydrolysis process [94]. For this purpose, there are different pre-treatment methods which exploit the use of chemicals: they involve acids, bases, ionic liquids, or organic solvents [95]. Despite this, the most used methods on an industrial scale are the physico-chemical ones, mainly steam explosion and hydrothermal pre-treatment. The first consists in subjecting HS to heating by using pressurized steam (150–300 °C, 1–5 MPa) for a few seconds or minutes; then, the reaction is stopped and there is an immediate return to atmospheric pressure [93,95]. This method causes structural degradation of lignin, making hemicellulosic xylans available for extraction. Steam explosion can be defined as a thermal, mechanical, and chemical process simultaneously. In fact, biomass degradation is caused by heat in the form of steam (thermal), by the shear forces due to the expansion of moisture (mechanical) and by the self-catalysed hydrolysis of glycosidic bonds (chemical) [96]. In particular, xylans are hydrolysed in XOS and xylose by organic acids, such as acetic acid, which in turn are formed due to the hydrolysis of functional groups (such as acetyl groups) linked to xylans [97]; for this reason, this technique is also called “autohydrolysis”. Time of pre-treatment, temperature, pH and moisture content are the parameters that most affect the efficiency of steam explosion pre-treatment [98,99]. This is an advantageous technique thanks to the non-use of chemicals with high environmental impact and to the low energy costs [93]. No literature studies about (A)XOS extraction through steam explosion were available. A more commonly used technique is hydrothermal pre-treatment. This is an operation in which the biomass is simply treated with water at high temperature. Generally, temperatures varying between 160 and 220 °C are used, in combination with high pressures for maintaining water at liquid state, for a period of about 15 min [93]. This technique leads to the hydrolysis of the xylan backbone, to the consequent depolymerization and therefore to (A)XOS solubilization. Here too, one can speak of autohydrolysis, because the hydrolysis of acetyl groups along the backbone (with consequent acetic acid formation) is responsible for subsequent hydrolysis of glycosidic bonds. At the end of the treatment, cellulose and lignin will instead be found in the solid phase with few chemical modifications [14,42] and can be separated and reused for other applications [100]. During hydrothermal pre-treatment, particle size is particularly important when a small liquid-to-solid ratio is used: in this case, the use of big particles can lead to a deficient impregnation and solubilization, generating reduced extraction yields because of an incomplete hydrolysis [101]. Autohydrolysis conditions (time and temperature) have a great influence on xylan depolymerization, therefore on their solubilization and on (A)XOS extraction yield. By increasing treatment severity, in fact, it is possible to obtain such a strong hydrolysis that it can lead to XOS decomposition in xylose and its dehydration products [42]. Vice versa, when lignocellulosic biomass is subjected to hydrothermal pre-treatment in milder conditions, XOS are the main products found in liquid phase [100]. 

Table 1 shows several examples found in literature relative to (A)XOS extraction from different agri-food sources and with different conditions. For example, Rivas and colleagues have investigated the optimal process conditions for obtaining XOS from HS, finding the maximum solubilization (74% of xylans in raw materials) at 210 °C for 15 min. Extracted XOS had DP varying from 3 to 16 [15]. Since (A)XOS with greater effects are the ones with low DP [42,65], a further treatment could be necessary, for example with acids or enzymes [102]. In the study conducted by Surek and Buyukkileci, the greater XOS extraction yield from HS was equal to 62% of xylans in raw material: this result was obtained through a hydrothermal pre-treatment at 190 °C for 5 min [14]. With these conditions, less than half of extracted XOS had low DP (2–6); though, by increasing the temperature, a great extraction yield was obtained but xylans were mainly degraded to xylose, furfural, and acetic acid [14]. Authors in this study have stressed that the time-temperature combination is fundamental and should be studied on a case-by-case basis. Moreover, holding time does not have the same effect on the extraction yield at all temperatures. In particular, it is necessary to also regulate autohydrolysis conditions in relation to desired DP [14]. In the study by Nabarlatz et al., a hydrothermal treatment at 179 °C for 23 min was tested on various agricultural by-products, obtaining a XOS yield ranging between 30 and 60% [103]. Ho et al., on the other hand, tested higher temperatures for a short time on oil palm empty fruit bunches fibre, obtaining a good yield (63%), but a rather high DP [104]. The same extraction yield was also obtained in another study on almond shells [105] (Table 1). In choosing the conditions of the process to be applied, the substituents on the xylan backbone, like acetyl and uronic groups, must also be considered. In fact, they have an influence on the solubility of XOS and their prebiotic effect [62] and therefore the sensibility of different functional groups to autohydrolysis must also be assessed. Arabinose, an essential constituent of AXOS, seems to degrade earlier than xylose [14], and acetyl groups, if hydrolysed, can lead to the formation of acetic acid in solution with consequent lowering pH, promoting further hydrolysis of xylan backbone [103]. As regards AXOS, whose extraction is more complicated compared to XOS due to the need to preserve arabinose, there are no studies in literature that deal with their extraction from HS. However, their extraction from cereals has been recently studied. As can be observed in Table 1, some research about AXOS extraction from nixtamalized maize pericarp and brewers’ spent grain have agreed on obtaining the best extraction yield at a temperature close to 210 °C [106,107]. Immerzeel and colleagues, on the other hand, found a decrease in the extraction yield and also in the degree of arabinose substitution from 195 °C on in the wheat bran [108]. Carvailhero et al. proposed the use of a temperature of 215 °C for a short time to obtain an AXOS yield of 64% from wheat straw, even if no information is available on the DP of the products obtained [109].

Ultimately, there are many factors that may influence the chemical composition of the raw material, such as genetics, growth area, or storage conditions [110], and consequently also the autohydrolysis conditions must be regulated time after time. However, there is no doubt that hydrothermal pre-treatment is a very valuable method for the extraction of these bioactive components. Indeed, this is a green technique, with relatively low costs and, unlike acid or alkaline based methods, it does not require specific corrosion-resistant materials [93,95]. However, autohydrolysis pre-treatments have a limit. Indeed, when (A)XOS are extracted through hot liquid water or steam explosion, as a result there is the appearance of numerous other compounds in the reaction media, in more or less large quantities: monosaccharides, furfural or hydroxymethylfurfural, acetic acid, protein-derived products, inorganic compounds, or other products derived from the extractive and acid-soluble lignin fractions of the feedstock [42]. For this reason, if the aim is to employ the extract in the production of functional foods, it is necessary to purify it. Among the most efficient methods there are ultrafiltration and nanofiltration through a membrane, eventually coupled with double ion-exchange resin, as demonstrated by Vegas et al. [111]. Membrane filtration consists in simple size-based separation, in which the low molecular weight contaminants are permeated and the (A)XOS are retained. This technique is considered one of the most promising thanks to its low energy requirements, easy handling and potential use not only on a laboratory scale but also on an industrial one [15]. Several studies have investigated the use of this method on different agricultural by-products, such as peanut or almond shells [112,113], recovering up to 70% XOS and removing most of acetic acid and monosaccharides [114]. Other techniques have been also studied for (A)XOS purification. Among these are anion-exchange chromatography or size-exclusion chromatography [115,116], adsorption, or solvent extraction [15]. 

### 4.2. Enzymatic Treatment

Another potentially green and functional extraction method is the one that involves the use of enzymes on HS. The enzymes usable for (A)XOS production are mainly endo-β-1,4-xylanases, and ones that remove lateral groups, such as the acetyl group; β-xylosidases are also widely used for the hydrolysis of xylans, but they tend to produce monosaccharides rather than OS [100]. Addition of the enzyme should generally take place after an initial chemical treatment (often alkaline solutions are employed) or autohydrolysis treatment. Indeed, it seems that lignin presence is the main factor which limits the hydrolysis by cellulolytic and hemicellulolytic enzymes and for this reason its partial degradation with a pre-treatment is necessary [117]. When an enzymatic hydrolysis is carried out directly on lignocellulosic native biomass, less than 20% of cellulose-deriving glucose is released [118] and the same result is predictable as regards hemicellulose. Moreover, the particle size of the materials to hydrolyse and their solubility may strongly affect the enzyme activity, in inverse and direct proportion, respectively. Especially in the case of HS, the high amount of lignin and the low solubility of the substrate make the direct activity of the enzyme almost impossible. However, enzymatic hydrolysis is very appreciated because it does not impact the environment, does not require special equipment, and also because it does not lead to the formation of undesired compounds, unlike autohydrolysis methods [42]. Besides these reasons, the choice of an enzymatic hydrolysis after a pre-treatment could be due to the need to obtain (A)XOS with a lower DP. As regards the process, the enzyme can be added directly to the reaction medium, immobilized, produced in situ via microbial fermentation, or immobilized inside the biomass [100]. Many enzymatic tests have been performed on different raw materials, such as corncobs [77,119], almond shell [114], oil palm frond fibres [120], and wheat bran [108], with variable (A)XOS yields and variable DP, although most obtained XOS had a good percentage of DP 2–4. In the study conducted by Mathew et al., AX extracted from wheat bran have been subjected to enzymatic hydrolysis with four different xylanases; all of them have been able to originate XOS and AXOS, with different DP and substitutions [121]. Despite this, it seems that GH10 family xylanases are more efficient in producing shorter and more substituted (A)XOS than GH11 family [121,122] and therefore are better for the production of low DP prebiotic AXOS. The degree of substitution of arabinose on the xylan backbone (A/X) also has a strong influence on enzyme activity and thus on (A)XOS production. In fact, it has been reported that a lower A/X ratio favours hydrolytic activity of the enzymes, whether they belong to the GH10 or to GH11 family [123]. However, further studies on microorganisms and their xylanase production are necessary, as well as their application, to improve (A)XOS yields. Research should focus on the development of viable and economic technologies to be applied on HS, reducing productions costs, simplifying the production process, and increasing (A)XOS production yield in order to develop a wider market. Amorim and colleagues have expected an increase in the use of synthetic biology in the near future for the creation of microorganisms that could work as biological factories for the direct fermentation of lignocellulosic biomasses and (A)XOS production [49]. An AXOS-based production process through direct fermentation of agro-industrial by-products has already been tested: the authors stressed how this strategy makes the entire production process simpler, reducing it to a single step [124,125]. Indeed, this would allow the reduction of costs related to both enzyme purchase and to the high number of phases involved in the process, improving the overall performance of the process [49]. However, the feasibility of this process should be evaluated case by case and according to substrate. HS have a great quantity of xylans, but of lignin too; Amorim’s review suggests that direct fermentation is potentially suitable especially for substrates with a high xylan/lignin ratio [49]. In any case, there are not many studies in literature about HS valorisation through (A)XOS production. Therefore, research in this field should be strongly encouraged and different techniques should be tested and optimized in order to allow the maximum exploitation of a very precious by-product.

## 5. Characterization of Obtained (A)XOS

If the optimization of (A)XOS production and purification processes is a challenging operation, perhaps their characterization is even more so. The development of an analysis that allows their characterization is essential, since (A)XOS nutritional and technological properties are strictly related to their structure. In this sense, the most influential factors are the DP, the stereochemistry of monosaccharides (xylose in the case of XOS, arabinose and xylose in the case of AXOS), the configuration of their bonds, the more or less extensive branching, and the type of substituents on the backbone [128]. Because of the need to determine all these aspects, it is clear that the structural analysis of carbohydrates turns out to be much more complex than that of other macromolecules, such as nucleic acids or proteins [129]. Despite the importance of carbohydrates, the current tools for their detailed structural characterization leave something to be desired: they have low reproducibility, low sensibility and specificity, and lack speed [128]. To date, in fact, there is no official method for analysing and quantifying OS or polysaccharides. Over the years, many different techniques have been developed, of varying efficiency. For some of them, the initial step involves an OS depolymerization leading to monosaccharides, that is followed by a chromatographic separation and finally an identification analysis. The first one is a delicate phase: although several depolymerization methods have been found, such as acid hydrolysis, enzymatic hydrolysis or methanolysis, it seems that each polysaccharide requires different optimized conditions [130]. When neutral monosaccharides are present, as in the case of (A)XOS, the acid hydrolysis is probably the more efficient method: in a three-method comparative study, this has been the one that has allowed the release of the greatest quantity [131]. Regarding separative analysis of originated monosaccharides, one of the most employed techniques has been gas chromatography coupled with mass spectrometry detector (GC-MS) or flame ionization detector (GC-FID). The first in particular has often been used not only for characterizing the monosaccharides originated from hydrolysis, but also the OS up to 2500 Da after permethylation [132]. Current platforms however have limited scope and are neither quick nor sufficiently sensitive [133,134]. Moreover, GC method requires analytes with high volatility, which is why derivatization techniques are needed, for example using methylation or acetylation [134]. This additional phase is not welcome because of the costs associated with the purchase of reagents and because it lengthens the analysis time. This does not happen when anion exchange chromatography, coupled with pulsed amperometric detector (HPAEC-PAD), is employed. This is another technique used in OS characterization and it offers specific and sensitive detection, as well as good OS separation [135]. In a recent study, this technique has allowed the separation and the quantification of XOS and AXOS, with a DP up to 6, mixed together [136], however it has some limits, such as the progressive loss of detector signal during the analysis, the expensive replacement of disposable electrodes, and the difficulty in separating and characterizing branched OS [135]. In particular, in AXOS analysis, it has been observed that the position of substituent arabinose affects the retention time, and that some of these AXOS can elute simultaneously, which complicates their identification [137]. Furthermore, according to some scholars, HPAEC-PAD could perhaps lead to epimerization and OS degradation when an aqueous solution with a high concentration of sodium hydroxide is used as the mobile phase [138]. Several studies have also tried to establish a method for OS structural identification by relying on liquid chromatography, coupled with different detectors. UV-vis detector has been employed in some cases, but it has the limit of needing a chromophore-based derivatization step, since monosaccharides do not have UV activity [128]. By contrast, HPLC-RID has permitted a good XOS identification in some studies, but limited to those with a low DP (2–4) [139,140]. RID is commonly used for the detection of OS and polysaccharides in general, especially when it is coupled with size-exclusion chromatography (HPSEC). The latter is widely employed because it provides information on molecular weight distribution and is particularly advantageous since it needs aqueous solvents, allowing for quick and easy sample preparation [141]. In fact, HPSEC separation principle is based on the hydrodynamic volume of molecules, which can be strongly influenced by the number and the length of any eventually present branch: therefore, molecules with the same hydrodynamic volume can have different molecular weights and, despite this, coelute [142]. For this reason, although sometimes this problem can be solved through the use of HPSEC with multiple-detection (by two independent methods, viscometry and light scattering), this technique can yield molecular weights with highly variable accuracy [142]. Bowman and colleagues have studied and well characterized the structure of AXOS originated from enzymatic hydrolysis, by reverse phase-high pressure liquid chromatography coupled with tandem mass spectrometry (RP-HPLC/MS-MS) after derivatization [143]. In general, positive ion MS/MS can be very useful for determining the position of the bonds and the monosaccharides sequence but can cause problems when there are same-type monomers, as in the case of (A)XOS [122]. In this sense, in fact, a derivatization step or a labelling step at the non-reducing end is necessary [144]. One limitation of MS is that it alone cannot discriminate between hexose isomers or pentose isomers, since they all have the same molecular formula and therefore the same mass [145]. Pu et al. proposed another method to characterize XOS, based on hydrophilic interaction liquid chromatography with evaporative light scattering detection (HILIC-ELSD), without the need for derivatization [138]. The coupling of HILIC with ELSD and with the MSn detector can give important structural information, such as the presence of acetyl groups, and allows the characterization and quantification of many OS with different structures; however, isomeric structures tend to coelute, leading to overlapping peaks [146]. In another study, neutral deprotonated AXOS structure was characterized through negative electrospray with quadrupole and time of flight coupled with mass spectrometer (ESI-Q-TOFMS) and through negative electrospray associated with ion trap and MS (ESI-ITMS): these techniques have proven to be particularly efficient for structural analysis of AXOS up to DP 9, including isomer differentiation. In reality, the interpretation of the spectra obtained by ESI-Q-TOFMS was successful also thanks to the knowledge gained from previous characterization through ^1^H NMR [147]. Nuclear magnetic resonance (NMR) is another effective tool for determining carbohydrates structures, especially monosaccharides composition, their configuration and sequence and the characteristics of the bond [134]. It has often been coupled with negative ESI-MS/MS and methylation analysis for the identification of new OS [129]. Its coupling with GC has been employed too, for analysing the nature of monosaccharides and the position of polysaccharide bonds, but these techniques are slow and often suffer from low sensibility [133]. In a recent study, Xiao and colleagues have extracted XOS from bamboo by autohydrolysis, then they separated and purified them by gel permeation chromatography (GPC) and finally characterized them by combining ESI-MS, NMR and HPAEC-PAD; the combination of ^1^H, ^13^C, and 2D HSQC NMR has given important structural information, especially on the sites where substitutions took place [148]. Another technology that has recently taken hold to study polysaccharide structure is matrix-assisted laser desorption/ionization coupled with mass spectrometry (MALDI-MS). It has proven to be an effective tool for determining (A)XOS molecular weights [122], and thanks to the ease in sample preparation and high speed of analysis, it is often used for offline MS analysis to identify DPs and the composition of separate oligomers [149]. Moreover, it has low fragmentation, large mass range and tolerance to impurities, and does not require derivatization [129], as shown in a study conducted on XOS from olive pulp [150]. More recently, however, more advanced techniques have been developed, such as MALDI-TOF-MS and MALDI post-source decay TOF/MS, which are up to ten times more sensitive [129]. MALDI-TOF-MS, in particular, has been employed for characterizing more or less substituted (A)XOS with DP ranging from 4 to 19 in peanut shells [112], wheat bran [151], birch wood [152], olive tree pruning by-products [153], and other agricultural residues [154]. Despite the fact that this technique allows a good determination of molecular masses, it is limited by not directly distinguishing the anomers and OS branched configurations [129]. Moreover, Fourier transform ion cyclotron resonance mass spectrometry can also be used to identify OS: this tool can give detailed information on their composition [155,156] and it has been used also for thioxylo-oligosaccharide determination [157]. It has important features, such as high resolution and high mass accuracy, which can help overcome many of the difficulties in the analysis of these compounds. However, complete structural elucidation may not be possible with mass spectrometry alone, but may require other tools, such as NMR and chemical and enzymatic methods [158]. Finally, ion mobility (IM) is a very promising technique in contributing to OS identification; it is often coupled with MS and allows ion separation based on their mass, charge, size, and shape, thus also allowing differentiation of isomers [159]. The principle on which IM is based is the separation of the analytes in a long drift tube, in which an electric field is applied and in which an inert gas passes, before they reach the detector for mass analysis; drift times, which vary according to the size and shape of the analytes, can be combined with MS spectra and integrated into databases for future structural identification [160]. The major advantages given by this technique are the high-resolution power, the high sensitivity, and the very low time required for the analysis. On the other hand, IM-MS is not yet powerful enough to separate almost identical structures: overlaps between species with similar drift time can in fact still occur [160]. Moreover, there are no studies in literature about (A)XOS structural characterization by IM-MS, but it is very likely that this technique will be in the foreground in the near future. Indeed, some studies have already shown a very high potential on the separation and discrimination between different OS [161]. 

## 6. Conclusions

Hazelnut shells are by-products with a very high potential that could create, if properly exploited, a huge added value. In fact, their high content of xylans and arabinoxylans in their hemicellulosic fraction creates the condition for obtaining prebiotic (A)XOS that may be re-used in the creation of functional foods, whose market is growing enormously. This review has been conceived to summarize the current knowledge about chemical structure of hazelnut shells’ (A)XOS, their potential health-promoting effects and the (A)XOS conditions of use as food ingredients. Moreover, a focus has been made on the main methods that have been developed to optimize (A)XOS extraction and purification steps, but also their structural characterization. In the latter field, in particular, the scientific community should concentrate its efforts in the near future, since it is the most complex issue and because we are still far from devising a technique which gives full-access information on the molecules of interest. To elucidate these structures in detail would allow us to study and further understand the relationship between the structure itself and the beneficial effects of (A)XOS. Finally, more studies on the industrial production of these compounds should be conducted, possibly with energy balance. As a result, it would be finally possible to take full advantage of HS, a waste product of the food industry that could become a treasure.

## Figures and Tables

**Table 1:** Different methods for (A)XOS extraction from different sources, consequent extraction yields, and DP obtained.

XOS/AXOS	Pretreatment/Extraction	Hydrolysis	Substrate	(A)XOS Production Yield	DP	Reference
xos	alkali	enzymatic hydrolysis	corncobs	81% of the original xylan	2–7	[77]
xos	acid	enzymatic hydrolysis	corncobs	52% of the original xylan	2–7	
xos	steam explosion	enzymatic hydrolysis	corncobs	77% of the original xylan	2–7	
xos	alkali	acid hydrolysis (H_2_SO_4_)	tobacco stalk	13% of the original xylan	1–6	[126]
xos	alkali	acid hydrolysis (H_2_SO_4_)	cotton stalk	7.5% of the original xylan	1–6	
xos	alkali	acid hydrolysis (H_2_SO_4_)	sunflower stalk	12.6% of the original xylan	1–6	
xos	alkali	acid hydrolysis (H_2_SO_4_)	wheat straw	10.2% of the original xylan	1–6	
xos	alkali	enzymatic hydrolysis	corncob	17.9% of raw material	2–5	[119]
xos	hydrothermal, 210 °C 15 min	-	hazelnut shell	73.7% of the original xylan	3–16	[15]
xos	hydrothermal, 190 °C, 5 min	-	hazelnut shell	62% of the original xylan	2–>6	[14]
xos	hydrothermal, 190 °C, 19 min	-	almond shell	63% of the original xylan	n.d	[105]
xos	hydrothermal, 179 °C, 23 min	-	corncobs	60% of the original xylan	n.d	[103]
xos	hydrothermal, 179 °C, 23 min	-	almond shell	55% of the original xylan	n.d	
xos	hydrothermal, 179 °C, 23 min	-	olive stones	43% of the original xylan	n.d	
xos	hydrothermal, 179 °C, 23 min	-	rice husks	30% of the original xylan	n.d	
xos	hydrothermal, 179 °C, 23 min	-	wheat straw	43% of the original xylan	n.d	
xos	hydrothermal, 179 °C, 23 min	-	barley straw	43% of the original xylan	n.d	
xos	hydrothermal, 210 °C, until reaching temperature then fast cooling	-	Palm empty fruit bunches fibre	63% of the original xylan	5–40	[104]
xos	hydrothermal, 121 °C, 60min	enzymatic hydrolysis	oil palm frond fibres	15% of raw material	1–4	[120]
xos	hydrothermal, 200 °C, 5 min	enzymatic hydrolysis	almond shell	54.5% of the original xylan	76.8% low DP	[114]
xos	steam explosion	enzymatic hydrolysis	barley straw	60.2% of the original xylan	4–9	[127]
axos	hydrothermal, 210 °C, 2 min	-	brewery spent grain	43.4% of the original AX	8–18	[106]
axos	hydrothermal, 207 °C, until reaching temperature then fast cooling	-	nixtamalized maize pericarp	39.6% of raw material (based on dm)	n.d	[107]
axos	hydrothermal, 185 °C, 10 min	enzymatic hydrolysis	wheat bran	59% of the original AX	2–5	[108]
axos	hydrothermal, 215 °C, until reaching temperature then fast cooling	-	wheat straw	64% of the original AX	n.d	[109]

## Data Availability

This study did not report any data.
